# Studying How Individuals Who Express the Feeling of Loneliness in an Online Loneliness Forum Communicate in a Nonloneliness Forum: Observational Study

**DOI:** 10.2196/28738

**Published:** 2021-07-20

**Authors:** Anietie Andy

**Affiliations:** 1 Penn Medicine Philadelphia, PA United States

**Keywords:** loneliness, Reddit, nonloneliness, mental health, eHealth, forum, online forum, communication, natural language processing, language, linguistics

## Abstract

**Background:**

Loneliness is a public health concern, and increasingly, individuals experiencing loneliness are seeking support on online forums, some of which focus on discussions around loneliness (loneliness forums). Some of these individuals may also seek support around loneliness on online forums not related to loneliness or well-being (nonloneliness forums). Hence, to design and implement appropriate and efficient online loneliness interventions, it is important to understand how individuals who express and seek support around loneliness on online loneliness forums communicate in nonloneliness forums; this could provide further insights into the support needs and concerns of these users.

**Objective:**

This study aims to explore how users who express the feeling of loneliness and seek support around loneliness on an online loneliness forum communicate in an online nonloneliness forum.

**Methods:**

A total of 2401 users who expressed loneliness in posts published on a loneliness forum on Reddit and had published posts in a nonloneliness forum were identified. Using latent Dirichlet allocation (a natural language processing algorithm); Linguistic Inquiry and Word Count (a psycholinguistic dictionary); and the word score–based language features *valence*, *arousal*, and *dominance*, the language use differences in posts published in the nonloneliness forum by these users compared to a control group of users who did not belong to any loneliness forum on Reddit were determined.

**Results:**

It was found that in posts published in the nonloneliness forum, users who expressed loneliness tend to use more words associated with the Linguistic Inquiry and Word Count categories on sadness (Cohen *d*=0.10) and seeking to socialize (Cohen *d*=0.114), and use words associated with *valence* (Cohen *d*=0.364) and *dominance* (Cohen *d*=0.117). In addition, they tend to publish posts related to latent Dirichlet allocation topics such as relationships (Cohen *d*=0.105) and family and friends and mental health (Cohen *d*=0.10).

**Conclusions:**

There are clear distinctions in language use in nonloneliness forum posts by users who express loneliness compared to a control group of users. These findings can help with the design and implementation of online interventions around loneliness.

## Introduction

Loneliness is a public health challenge [1]; it affects the well-being of individuals of all age groups [2,3] and has been linked to early death [4,5], depression [6,7], and heart disease [8].

Several prior works [1,9,10] have analyzed data from social media platforms and online forums, some of which focus on discussions around loneliness (loneliness forums) to understand the support needs of individuals who express loneliness on these platforms. These individuals may also seek support and express concerns as it relates to loneliness, such as how to develop or maintain relationships, on online forums not focused on discussions around loneliness or well-being (nonloneliness forums). To better understand the support needs of users who express loneliness on online loneliness forums and to design and implement appropriate and efficient online interventions, it is important to study how these individuals communicate in nonloneliness forums.

Using natural language processing methods, prior works determined that the language used in posts published on social media platforms and online forums can be used to gain insights into how users communicate on these forums and the types of support they seek and express, as it relates to their health and well-being. For example, language used in posts published on Facebook was used to train a machine learning model to predict patients risk for cardiovascular disease [11]. In addition, language used on online forum posts were used to determine the support needs of users in substance use recovery forums [12], a COVID-19 online forum [13,14], and an online cancer forum [15-17]. Regarding loneliness, prior works analyzed social media data belonging to individuals who expressed loneliness; for example, Guntuku et al [1] analyzed Twitter posts from users who self-declared feeling lonely, and it was determined that the language used by these users in their Twitter posts was more associated with mental health concerns. Kivran-Swaine et al [18] determined that posts in which users expressed loneliness tended to receive more responses compared to other posts by the same users. Ruiz et al [9] showed that the more loneliness users express on social media, the less online relationships they had. Hunt et al [10] showed that there is an association between less social media use and a decrease in the feeling of loneliness and depression.

Similar to prior works, in this study, using natural language processing methods, language used on posts published on a nonloneliness online forum are analyzed to gain insights into the support needs and concerns of individuals who express loneliness in a loneliness online forum. Potentially, information gleaned from the analyses in this study will provide further insights into the support needs and concerns of individuals expressing loneliness on online loneliness forums, thereby informing loneliness interventions.

## Methods

### Data and Design

For the analysis in this study, data from Reddit was used. Reddit is made up of more than 1 million subforums (called subreddits) [19] focused on discussions around specific topics such as depression, loneliness, and open-ended questions spanning various topics. In addition, Reddit allows members to join several of these forums; hence, it is possible to get posts published in a nonloneliness forum by users who are members of a loneliness forum. Using Google’s BigQuery [20], which is a data warehouse with publicly accessible Reddit data sets published between December 2015 and August 2019, posts from Reddit forums focused on discussions around loneliness were identified by selecting the forums that contained the word “Lonely” in its name (eg, “/r/lonelyheartbeats,” “/r/iAMlonely,” and “/r/Lonely”). It was observed that the forum */r/Lonely* had more posts and members compared to the other forums, which each had less than 200 published posts during the time period in which the data was collected. Hence, for the analysis in this study, usernames of members of */r/Lonely* were used; specifically, the usernames of 9956 users who had published a total of 15,012 posts on the */r/Lonely* forum were selected.

To identify the other Reddit forums in which these users belong to and tend to publish posts, using the usernames from */r/Lonely*, all the forums on Reddit were searched to determine the forums in which these */r/Lonely* users tend to publish posts. It was observed that the forums with the most number of these users as members are */r/AskReddit* (a forum in which users seek advice and ask open-ended questions on various topics) and */r/depression* (a forum focused on discussions around depression) with posts by 24% (n=2401) and 20% (n=2031), respectively, of the */r/Lonely* users (N=9956) in the data set. Since the focus of this study is to determine how individuals who express loneliness on an online loneliness forum communicate in nonloneliness forums (ie, forums not focused on loneliness or well-being), for all the analysis in this study, data from */r/AskReddit* by 2401 users who expressed loneliness in */r/Lonely* were used. The author reviewed the posts (N=4001) published on */r/Lonely* by these users and observed that these users expressed feeling lonely in their */r/Lonely* posts by stating that they were feeling lonely (eg, rephrased “I am a 25 years old female and I am always lonely”), implied that they were feeling lonely (eg, rephrased “I moved to a new city and I don’t know anyone”), or sought support as it relates to loneliness (eg, rephrased “Where can I find tools online to help with loneliness?”).

Each of the 2401 users who posted on */r/Lonely* and had published posts on */r/AskReddit* were matched with a control group user who had no published posts on any loneliness forum on Reddit and had published posts on */r/AskReddit* between December 2015 and August 2019.

Table 1 shows information about the data set.

**Table 1:** Summary of /r/AskReddit posts published between December 2015 and August 2019 by /r/Lonely users and a control group.

Variables	/r/Lonely users	Control group
Users, n	2401	2401
Posts, n	25,834	34,718

In this study, the following methods were used to determine language use differences in */r/AskReddit* posts by users who express loneliness compared to the control group: a topic modeling approach, a dictionary-based approach, and a word score–based approach. Cohen *d*, which indicates the standardized difference between means, was used to report the effect sizes. In this study, only results with Cohen *d* greater than or equal to a threshold (ie, 0.10) and that are significant at Bonferroni-corrected *P* values <.001 are reported.

The topic modeling approach and the dictionary-based approaches were used because prior works used these approaches to gain insights from social media data about the language use differences between individuals in different genders [21] and age groups [22], and to determine the language use differences between users who express loneliness compared to a control group of users who did not express loneliness [1]. The word score–based approach was used because prior work [23] used these methods to better understand language features associated with persuasion in online forum posts and comments.

### Topic Modeling Approach

In this section, the natural language processing topic modeling method latent Dirichlet allocation (LDA) [24] was used. LDA works by, first, splitting words in Reddit posts into single words or tokens (tokenization). Second, words that co-occur together are clustered together; the cluster of words are referred to as topics, and based on the content words associated with each topic, a label can be assigned to the topics. For example, LDA could group the words “family,” “mom,” “dad,” “daughter,” and “son” as a reference to family. LDA assumes that the topics consist of a combination of words, and each Reddit post is made up of a combination of topics. Using the Dlatk package [25], 20 LDA topics were generated from the */r/AskReddit* posts associated with */r/Lonely* users and the control group users; to determine the number of LDA topics, the number of topics varied between 5 and 50 topics by starting with 5 topics and incrementing by 2 topics up to 50 topics. A total of 20 topics had the most coherent topic themes when reviewed by the author. With the generated topics, using the Dlatk package [25], the topic themes that frequently occurred in the */r/AskReddit* posts by */r/Lonely* users when compared with the control group users were identified.

### Dictionary-Based Approach

In this approach, language from */r/AskReddit* posts associated with the */r/Lonely* users and the control group users were used to determine the prevalence of Linguistic Inquiry and Word Count (LIWC) [26] dictionary word categories in posts associated with these groups of users. LIWC is a psycholinguistic dictionary made up of 73 predefined categories such as positive and negative emotions; each of these categories has a curated list of words associated with it. LIWC has been used in several prior works [1,22,27]. Using the Dlatk package [25], for each group of */r/AskReddit* posts (ie, posts belonging to */r/Lonely* users and the control group users), the proportion of token words associated with LIWC categories were determined.

### Word Score–Based Approach

The word score–based features v*alence*, *arousal*, and *dominance* have been used by prior works to study communication strategies in an online forum [23,28]. *Valence* indicates the measure of the positive or negative denotation of a word; for example, “enjoyable” is a high *valence* word and “nightmare” is a low valence word. *Arousal* measures the emotional intensity expressed in a word; an example of a high *arousal* word is “exhilarated,” and an example of a low *arousal* word is “siesta.” Dominance indicates the measure of the locus of control expressed in a word; for example “powerful” is a high dominance word, and “weak” is a low dominance word. Mohammad [28] provided a lexicon of human ratings for *valence*, *arousal*, and *dominance* for 20,000 words in English; using this lexicon, for each post in the data set, the average ratings of all content words in each of these word categories (ie, *valence*, *arousal*, and *dominance*) was computed.

## Results

### Topic Modeling Approach

Table 2 shows the most significant LDA topics in */r/Askreddit* posts by */r/Lonely* users compared to the control group.

**Table 2:** Results from latent Dirichlet allocation analysis on /r/AskReddit posts by /r/Lonely users compared to the control group users.

Label	Highly correlated words	Cohen *d*	Mean (SD)
Relationships	people, love, hate, relationship, find, can't, lose, meet, stop, married	0.105	0.038 (0.048)
Family and friends/mental health	family, friends, deal, talk, experience, depression, mental, care, service, dear	0.10	0.038 (0.053)

### Dictionary-Based Approach

Table 3 shows the different LIWC categories in */r/AskReddit* posts most associated with */r/Lonely* users compared to the control group users.

**Table 3:** Results from LIWC analysis on /r/AskReddit posts by /r/Lonely users compared to the control group users.

LIWC^a^ category	Cohen *d*	Mean (SD)
Social processes	0.114	0.157 (0.08)
Sadness	0.10	0.083 (0.042)

^a^LIWC: Linguistic Inquiry and Word Count.

### Word Score–Based Approach

Using Cohen *d*, the effect size between the features that represent the average *valence*, *arousal*, and *dominance* scores for posts in the data set and a feature that represents if a */r/AskReddit* post was by a */r/Lonely* user or a control group user was determined, as shown in Table 4.

**Table 4:** Results from word score–based language feature analysis on /r/AskReddit posts by /r/Lonely users compared to the control group users.

Attribute	Cohen *d*
Valence	0.364
Arousal	–0.004
Dominance	0.117

## Discussion

### Findings

Using natural language processing methods, this study shows the distinction in language use in posts published on a nonloneliness forum by users who express and seek support around loneliness in an online loneliness forum compared to a control group of users. These language use differences reflect the support needs and concerns of these users. The findings from this study are summarized in this section.

This study determined that users who express the feeling of loneliness in */r/Lonely* tend to seek advice and ask questions about relationships on */r/AskReddit* (Table 2) compared to the control group users. This finding is in line with prior work [1] that determined that individuals who expressed loneliness on Twitter tend to publish Twitter messages related to themes about difficult interpersonal relationships.

In addition, this study has findings that were not in prior work; specifically, it was observed that individuals who express loneliness in a loneliness forum tend to seek advice and ask questions about mental health concerns as it relates to their family members and friends; for example, the following are examples of */r/AskReddit* posts (rephrased) by */r/Lonely* users seeking advice as it relates to their relationships with family members and friends:

I need advice on how to deal with a family member / friend who keeps criticizing me.

I need help, if one is struggling with mental health, what is the best way to explain it to family members and friends?

Using LIWC, it was observed that users who expressed loneliness in the loneliness forum tended to use more words associated with sadness and wanting to socialize in the nonloneliness forum.

Using the word score–based language features v*alence*, *arousal*, and *dominance*, it was determined that the average v*alence* and *dominance* scores in */r/AskReddit* posts are more associated with posts by users who express loneliness on */r/Lonely*. A potential explanation for *dominance* being more associated with */r/AskReddit* posts by */r/Lonely* users is that some of these users seek support and express vulnerability in these posts; low dominance words suggest vulnerability, hence the association. Additionally, a potential explanation for v*alence* being more associated with */r/AskReddit* posts by */r/Lonely* users is that these users tend to use low *valence* words in these posts, hence the association.

This study shows that users who express loneliness in a loneliness forum seek support and communicate differently from a control group of users in a nonloneliness forum. The findings from this study can aid in the design and implementation of online loneliness interventions; for example, given that users who express loneliness in the loneliness forum ask questions and seek advice as it relates to their relationships (with family and friends) and use more words associated with seeking to socialize and sadness (Table 3), online loneliness interventions can provide services in which advice and tips are given to users on how to develop, maintain, and navigate relationships.

From this study’s findings, when designing and implementing online loneliness interventions, it is important to not only focus on user communication in loneliness forums but also look into how these users communicate in nonloneliness forums.

### Limitations

In this study, data from Reddit users was used and may not be representative of all individuals (some of whom may not publish posts on online forums expressing their feeling of loneliness) who feel lonely.

### Ethics and Privacy

The data set used for this study is publicly available. For all the analyses in this study, no user or moderator of any loneliness forum on Reddit (including */r/Lonely*) was contacted. In addition, besides the usernames of */r/Lonely* users, no other information from user profiles was used or accessed.

### Conclusion

In this study, using natural language processing methods*,* it was determined that users who express loneliness in an online loneliness forum communicated differently in a nonloneliness forum when compared to a control group of users. The findings from this study can aid with the design and implementation of online loneliness interventions.

## Abbreviations

LDA: latent Dirichlet allocation
LIWC: Linguistic Inquiry and Word Count
